# In-hospital Heart Rate Reduction With Beta Blockers and Ivabradine Early After Recovery in Patients With Acute Decompensated Heart Failure Reduces Short-Term Mortality and Rehospitalization

**DOI:** 10.3389/fcvm.2021.665202

**Published:** 2021-07-30

**Authors:** Alessandro Faragli, Giuseppe Di Tano, Caterina De Carlini, Daniel Nassiacos, Mauro Gori, Giada Confortola, Francesco Paolo Lo Muzio, Konstantinos Rapis, Dawud Abawi, Heiner Post, Sebastian Kelle, Burkert Pieske, Alessio Alogna, Carlo Campana

**Affiliations:** ^1^Department of Internal Medicine and Cardiology, Campus Virchow-Klinikum, Charité – Universitätsmedizin Berlin, Berlin, Germany; ^2^Berlin Institute of Health, Berlin, Germany; ^3^DZHK (German Centre for Cardiovascular Research), Partner Site Berlin, Berlin, Germany; ^4^Department of Internal Medicine/Cardiology, Deutsches Herzzentrum Berlin, Berlin, Germany; ^5^Department of Cardiology Ospedale Maggiore, ASST Cremona, Cremona, Italy; ^6^Department of Cardiology, Merate Hospital, ASST-Lecco, Lecco, Italy; ^7^Department of Cardiology, Ospedale di Circolo, ASST Valle Olona, Saronno VA, Italy; ^8^Department of Cardiology, ASST Ospedale Papa Giovanni XXXIII, Bergamo, Italy; ^9^Department of Surgery, Dentistry, Paediatrics and Gynaecology, University of Verona, Verona, Italy; ^10^Department of Medicine and Surgery, University of Parma, Parma, Italy; ^11^Department of Cardiology, Contilia Heart and Vessel Centre, St. Marien-Hospital Mülheim, Mülheim, Germany; ^12^Department of Cardiology Sant'Anna Hospital, ASST-Lariana, Como, Italy

**Keywords:** heart failure, observational study, risk stratification, therapy, heart rate

## Abstract

**Objective:** In the past years, heart rate (HR) has emerged as a highly relevant modifiable risk factor for heart failure (HF) patients. However, most of the clinical trials so far evaluated the role of HR in stable chronic HF cohorts. The aim of this multi-center, prospective observational study was to assess the association between HR and therapy with HR modulators (beta blockers, ivabradine, or a combination of ivabradine and beta blockers) at hospital discharge with patients' cardiovascular mortality and re-hospitalization at 6 months in acutely decompensated HF patients.

**Materials and Methods:** We recruited 289 HF patients discharged alive after admission for HF decompensation from 10 centers in northern Italy over 9 months (from April 2017 to January 2018). The primary endpoint was the combination of cardiovascular mortality or re-hospitalizations for HF at 6 months.

**Results:** At 6 months after discharge, 64 patients were readmitted (32%), and 39 patients died (16%). Multivariate analysis showed that HR at discharge ≥ 90 bpm (*OR* = 8.47; *p* = 0.016) independently predicted cardiovascular mortality, while therapy with beta blockers at discharge was found to reduce the risk of the composite endpoint. In patients receiving HR modulators the event rates for the composite endpoint, all-cause mortality, and cardiovascular mortality were lower than in patients not receiving HR modulators.

**Conclusions:** Heart rate at discharge ≥90 bpm predicts cardiovascular mortality, while therapy with beta blockers is negatively associated with the composite endpoint of cardiovascular mortality and hospitalization at 6 months in acutely decompensated HF patients. Patients receiving a HR modulation therapy at hospital discharge showed the lowest rate of cardiovascular mortality and re-hospitalization.

## Introduction

Heart failure (HF) represents not only the most common cause of hospitalization in Europe (1) but also one of the leading causes of mortality worldwide (2). Moreover, HF prevalence is expected to grow exponentially in the next years, mainly due to the aging of the population (1). Hence, the identification of a correct prognostic assessment plays a central role in HF management (3). Multiple markers and risk scores have been shown effective in stratifying the risk of mortality and hospitalization in HF patients (3). Resting heart rate (HR) has been found to be an easy, cost-effective, and reliable parameter able to predict cardiovascular morbidity and mortality in HF patients, irrespective of their left ventricle ejection fraction (LVEF) (4–7). The protective action of beta blockers in patients with heart failure with reduced ejection fraction (HFrEF) is well-established (8), and the reduction of HR has been already demonstrated to improve survival (9, 10). However, optimal HR control with beta blockers is sometimes not achieved in patients with HF due to a handful of reasons, including poor tolerance, side effects, as well as difficulties in drug titration (9, 10). Therefore, additional HR reducing therapies have been investigated with preliminary evidence for ivabradine, which is effective to lower HR without major effects on blood pressure and myocardial function (11, 12). Following one of the major clinical studies, the SHIFT trial (13), ivabradine has currently reached a class IIa level B recommendation as a combination therapy with beta blockers in the ESC HF guidelines for chronic stable HF patients (14). Nonetheless, acutely decompensated HF patients still present an unacceptably high mortality rate during hospitalization (5–7%), with significant values at 1 year after the acute episode (20–25%), as compared to chronic HF patients 1 year after diagnosis (6%) (15, 16).

The HR decrease during hospitalization has been found to positively protect against the development of long-term events (17), with a lot of studies focusing on beta blockers only. On the contrary, the role of ivabradine in acute settings is still limited to small studies or even just case reports (7). With this study, we aimed at investigating the effect of HR modulating drugs, namely, beta blockers and ivabradine, during hospitalization and their prognostic impact at 6 months.

## Materials and Methods

The study was conducted according to the directions of the Declaration of Helsinki (1964) with the approval of the local Ethical Committees and acquiring patients' informed consent. A prospective, multicenter, observational study in which we investigated the incidence and the predictors of mortality or re-hospitalizations at 6 months among 289 consecutive HF patients discharged alive after admission for *de-novo* as well as HF worsening in eight hospitals in northern Italy over 9 months of recruitment between September 2017 and June 2018. Patients 18 years of age or older were eligible for inclusion independently of their left ventricular ejection fraction (LVEF) if they had an N-terminal pro-B-type natriuretic peptide (NT-proBNP) concentration of 1,000 pg per milliliter or more or a B-type natriuretic peptide (BNP) concentration of 200 pg/ml or more and had signs and symptoms of fluid overload. Patients were enrolled no <24 h and up to 10 days after initial presentation to the hospital, while they were still hospitalized. Patients were required to be hemodynamically stable, which was defined by the maintenance of systolic blood pressure of at least 100 mm Hg for the preceding 6 h with no increase in the dose of intravenous diuretics and no use of intravenous vasodilators during the preceding 6 h and no use of intravenous inotropes during the preceding 24 h. Furthermore, patients with severe cognitive decline as well as with a prior cancer diagnosis with a limited prognosis (below 6 months) were excluded. The primary endpoint was a combination of cardiovascular death or re-hospitalizations for HF. The follow-up examination at 6 months was performed in the outpatient clinic of each hospital; in specific cases of patients with poor compliance or those not able to come back to the hospital, a telephone follow-up was scheduled.

### Definitions

Since the enrolled patients presented multiple comorbidities and various parameters at admission, we provide here the following definitions. Heart rate modulators were defined as the presence of either single or combination therapies with beta blockers and/or ivabradine. RV dysfunction was defined as fractional area change (FAC) <30%; history of revascularization during the hospitalization was defined as any type of coronarography or stenting performed during the hospital stay; symptomatic peripheral arterial occlusive disease (PAOD) was defined as intermittent claudication after 100 m or presence of critical limb ischemia (rest foot pain, ulcers, or gangrene); diabetes was defined as fasting plasma glucose ≥126 mg/dl; frailty was defined as a clinical syndrome in which three or more of the following criteria were present: unintentional weight loss (5 kg in past year), self-reported exhaustion, weakness (grip strength), slow walking speed, and low physical activity (18); anemia was defined as a hemoglobin concentration <7.5 mmol/L (12 g/dl) in women and <8.1 mmol/L (13 g/dl) in men.

### Evaluation of the Effects of HR Modulators Therapy

The effects of HR modulation therapy were studied during hospitalization and at 6 months follow-up. The main aim was to investigate the prognostic impact of HR modulators. This was assessed by comparing event rates at follow-up in the different observed groups. Event rates were calculated as the number of subjects on each HR modulation therapy divided by the total number of observed patients in the three considered endpoints: mortality, cardiovascular mortality, and composite endpoint.

### Statistical Analysis

Data were analyzed using Microsoft Excel and IBM SPSS Statistics version 23.0 software (SPSS Inc., Chicago, IL, USA) for Windows. Variables at admission and at discharge are presented as frequencies and percentages for binary variables or means and standard deviations for quantitative variables (median and interquartile range for NT-proBNP). A *p*-value < 0.05 was considered significant. Selected parameters at admission and at discharge were entered in a logistic regression univariable analysis. Baseline characteristics significantly associated with the endpoints (threshold for *p*-values < 0.1) by univariable analysis were entered as candidate variables in a multivariable logistic regression analysis. The final multivariable model was selected using a backward-elimination algorithm (retention threshold *p* < 0.05). We then repeated the analysis by stratifying the population based on the age. We set the threshold at >75 years, assuming it as a predetermined risk factor of mortality for HF patients, as already described in literature (19, 20). We assumed the threshold of HR > 90 bpm as a candidate risk predictor for major adverse events based on previous literature (21, 22).

## Results

**Figure 1:**
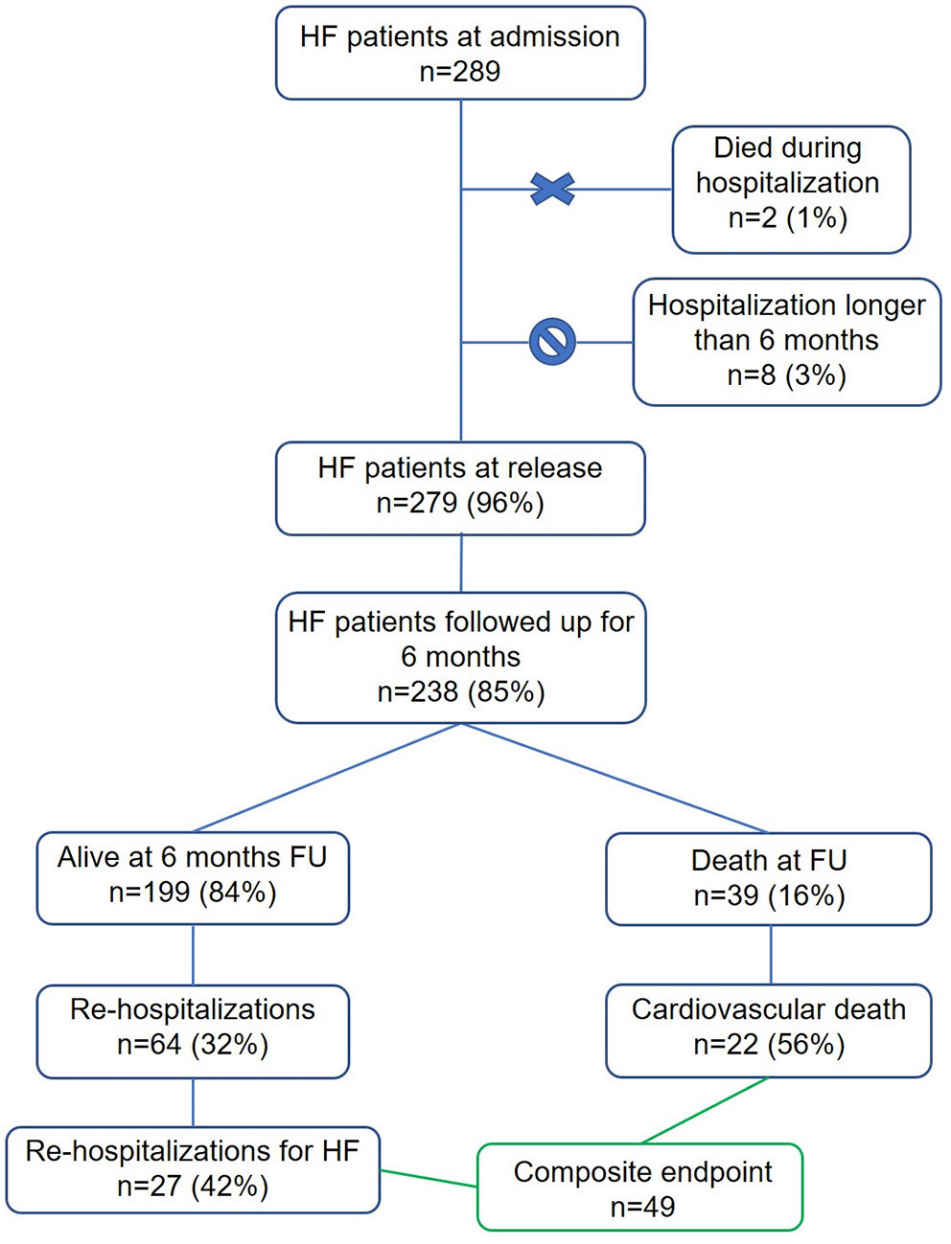
Study profile. In green are highlighted the 49 patients included in the created composite endpoint of re-hospitalization for HF and cardiovascular death.

A total of 289 patients were consecutively admitted to either cardiological or internal medicine departments of the above-mentioned hospitals with a diagnosis of acutely decompensated HF. Of the admitted patients, 23 (8%) presented as NYHA class II, 252 (89%) as NYHA class III, while 8 (3%) presented as NYHA class IV. The main HF-etiology was ischemic in 44%, cardiomyopathy-related in 17%, valvular in 15%, hypertensive in 13%, and arrhythmic in 11%. Of the admitted patients, two died during the hospitalization, while eight patients had a hospitalization longer than 6 months and were excluded from the final database. A total of 238 patients were followed up at 6 months after discharge (Figure 1). Patients were discharged with the following rates of HF therapy: ACE-I 54%, ARBs 33%, diuretics 68%, MRA 63%, calcium antagonists 15%, and amiodarone 24%.

### Baseline Characteristics and Changes in Clinical Variables

The characteristics of the patients at admission are summarized in Table 1. Patients had multiple comorbidities: 123 had diabetes (43%), 38 neoplasia (13%), 45 dysthyroidism (16%), 72 chronic obstructive pulmonary disease (25%), 6 asthma (2%), 21 symptomatic peripheral arterial disease (7%), 121 chronic kidney disease stage III (42%), 7 osteoarthritis (2%), and 66 frailty (23%).

**Table 1:** Population characteristics and HR modulator therapy received.

**Baseline characteristics**	**Total**	**No HR modulators**	**HR modulators**	***p*-Value**
	***n* = 289**	**(*n* = 114)**	**(*n* = 175)**	
Males (%)	169 (58)	107 (63)	62 (37)	0.155
Age (*SD*)	76 ± 10	76 ± 12	77 ± 10	0.766
Average length of hospitalization (*SD*)	16 ± 12	17 ± 12	16 ± 13	0.547
HF *de novo* (%)	132 (47)	69 (61)	63 (36)	<0.001
HF worsened (%)	154 (53)	44 (38)	110 (63)	<0.001
LVEF (*SD*)	39 ± 12	40 ± 12	38 ± 12	0.310
HFrEF (%)	219 (78)	83 (76)	136 (79)	0.333
RV dysfunction (%)	64 (23)	20 (18)	46 (26)	0.055
Afib (%)	121 (42)	47 (41)	74 (43)	0.462
ICD (%)	19 (7)	3 (3)	16 (9)	0.022
CRT/CRT-D (%)	23 (8)	5 (4)	18 (10)	0.053
Revascularization (%)	50 (18)	13 (12)	37 (21)	0.028
Peripheral congestion (%)	232 (81)	88 (79)	144 (82)	0.264
Diabetes (%)	123 (43)	42 (38)	81 (46)	0.089
Neoplasia (%)	38 (13)	15 (13)	23 (13)	0.543
Dysthyroidism (%)	45 (16)	14 (13)	31 (18)	0.162
COPD (%)	72 (25)	23 (21)	49 (28)	0.099
Asthma (%)	6 (2)	2 (2)	4 (2)	0.565
Symptomatic PAOD (%)	21 (7)	8 (7)	13 (7)	0.562
CKD with GFR <50 (%)	121 (42)	37 (33)	84 (48)	0.007
Ostheoarthritis (%)	7 (2)	3 (2)	4 (2)	0.560
Fraility (%)	66 (23)	24 (21)	42 (24)	0.361
Anemia (%)	75 (26)	32 (29)	43 (25)	0.268
Hyponatremia (%)	17 (6)	7 (6)	10 (6)	0.521

*Quantitative data are presented as mean ± SD, while binary variables are reported as frequencies and percentages in parentheses. The p-value for each feature is reported*.

Over half of the patients had worsening HF, the others had *de-novo* HF. The average LVEF of the whole population was 39 ± 12%, and patients with HFrEF were the majority (78%). Atrial fibrillation (AFib) was present in 121 patients (42%), while some patients bore implantable devices, either ICD, as in 19 patients (7%), or either a CRT or CRT-D device, as in 23 patients (8%). A right ventricular dysfunction was found in 64 patients (23%). The average length of hospitalizations was of 16 ± 12 days.

### Heart Rate Modulators

By stratifying the population into two groups, without HR modulator therapy, and with HR modulator therapy, meaning either beta blocker or ivabradine or a combination of the two, we identified the following differences in basic characteristics (Table 1). More specifically, we significantly found more patients hospitalized with a *de novo* HF in the group administered with no HR modulators, while patients already on HR modulators were generally hospitalized for worsened HF. Moreover, in the group treated with HR modulators, we found more patients were carrying an ICD or were implanted during the hospitalization. Moreover, we significantly found more patients that underwent revascularization during the hospitalization in the group treated with HR modulators. Interestingly, the group of patients with HR modulators had also a CKD with GFR <50%, underlining a higher severity of this population group.

At discharge, mean HR, systolic and diastolic blood pressure, body weight, and median NT-proBNP dropped as a sign of successful re-compensation (Table 2).

**Table 2:** Blood pressure, weight, heart rate, and NT-proBNP at admission and discharge.

**Parameters**	**Admission**	**Discharge**	***p*-Value**
Systolic blood pressure (*SD*) (*n* = 273) vs. (*n* = 266)	132 ± 25	118 ± 17	<0.001
Diastolic blood pressure (*SD*) (*n* = 273) vs. (*n* = 266)	76 ± 14	69 ± 9	<0.001
Heart rate (SD) (*n* = 275) vs. (*n* = 267)	86 ± 24	70 ± 12	<0.001
Weight (SD) (*n* = 243) vs. (*n* = 214)	74 ± 20	69 ± 19	<0.001
NTproBNP [IQ] (*n* = 227) vs. (*n* = 227)	2,989 [860–8,654]	1,200 [412–3,725]	<0.001

*Data are presented as mean ± SD, while NT-proBNP is presented as median and interquartile range. p-value < 0.05.*.

At admission, 170 (59%) patients were treated with beta blockers only, 17 (6%) with ivabradine only, and 12 (4%) were on a combination of the two drugs, accounting for a total of 187 (65%) of patients on at least one HR modulator (Table 3). At discharge, the number of patients on beta blockers were 221 (79%), on ivabradine 38 (14%), and 30 (13%) on a combination therapy of the two, accounting for a total of 259 (93%) on at least one HR modulator (Table 3). At follow-up, the number of patients on beta blockers was 148 (62%), the ones on ivabradine 35 (15%) and on a combination of the two drugs 28 (12%), accounting for a total of 183 on at least one HR modulator (77%) (Table 3).

**Table 3:** Hart rate at admission, discharge, and follow-up stratified for patients not receiving heart rate modulators vs. the ones receiving heart modulators.

	**No heart rate modulators (%)**	**Mean heart rate (*SD*)**	**Heart rate modulators (%)**	**Mean heart rate (*SD*)**
Admission *n* = 289	102 (35)	92 ± 27	187 (65)	82 ± 20
Discharge *n* = 279	20 (7)	71 ± 13^*^	259 (93)	69 ± 10^*^
Follow-up *n* = 238	55 (23)	69 ± 12^*^	183 (77)	69 ± 10^*^

*Data are presented as the total number of patients with or without heart rate modulators and their respective percentage to the population. Data regarding heart rate are presented as mean ± SD*.

* *p-value < 0.05 vs. admission*.

**Figure 2:**
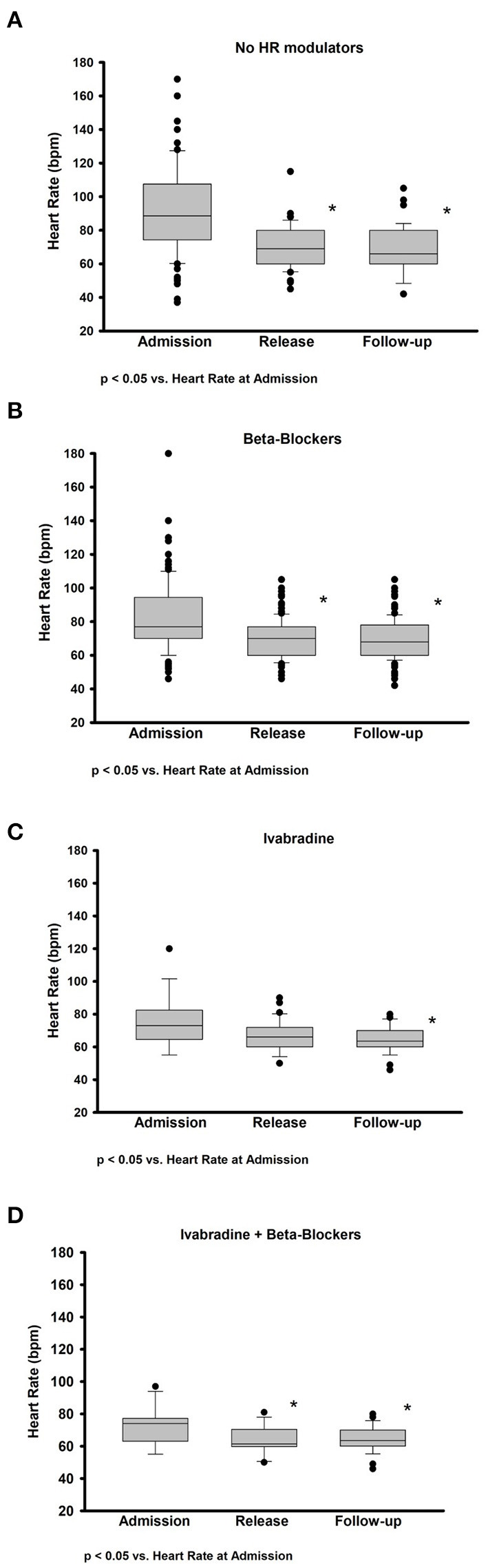
Impact on heart rate of therapy with and without heart rate modulators at admission, discharge, and follow-up. Box plots representing the absolute change in HR between admission, discharge, and follow-up after therapy with **(A)** no HR modulators, **(B)** only beta blockers, **(C)** only ivabradine, and **(D)** a combination of ivabradine and beta blockers (Ivabradine + Beta blockers). Data are presented as median and interquartile range. *p-value < 0.05.

The impact of HR modulators on HR is presented graphically in Figures 2A–D, divided by the type of HR modulator. The group of patients not treated with HR modulator had the highest HR at admission, however, a significant decrease in HR was achieved during admission and at follow-up (Figure 2A). In the subgroup treated with beta blockers a significant decrease in HR was observed during the hospitalization period, however, HR remained unchanged at follow-up (Figure 2B). Ivabradine alone, instead, did not significantly reduce HR during the hospitalization, while it did so at follow-up (Figure 2C). Finally, in the patients treated with both beta blockers and ivabradine, a significant decrease in HR was observed during hospitalization, which was maintained at follow-up (Figure 2D). When dividing the patients between the ones affected by Afib and the ones with sinus rhythmus, we observed a significant difference in the HR at discharge. The average HR at discharge of patients with AFib was 73 ± 11 bpm, while the average HR of patients without AFib was 68 ± 12 bpm (*p* < 0.001). None of the patients with AFib was treated with ivabradine.

### Event Rates at Follow-Up According to Heart-Rate Modulators

**Figure 3:**
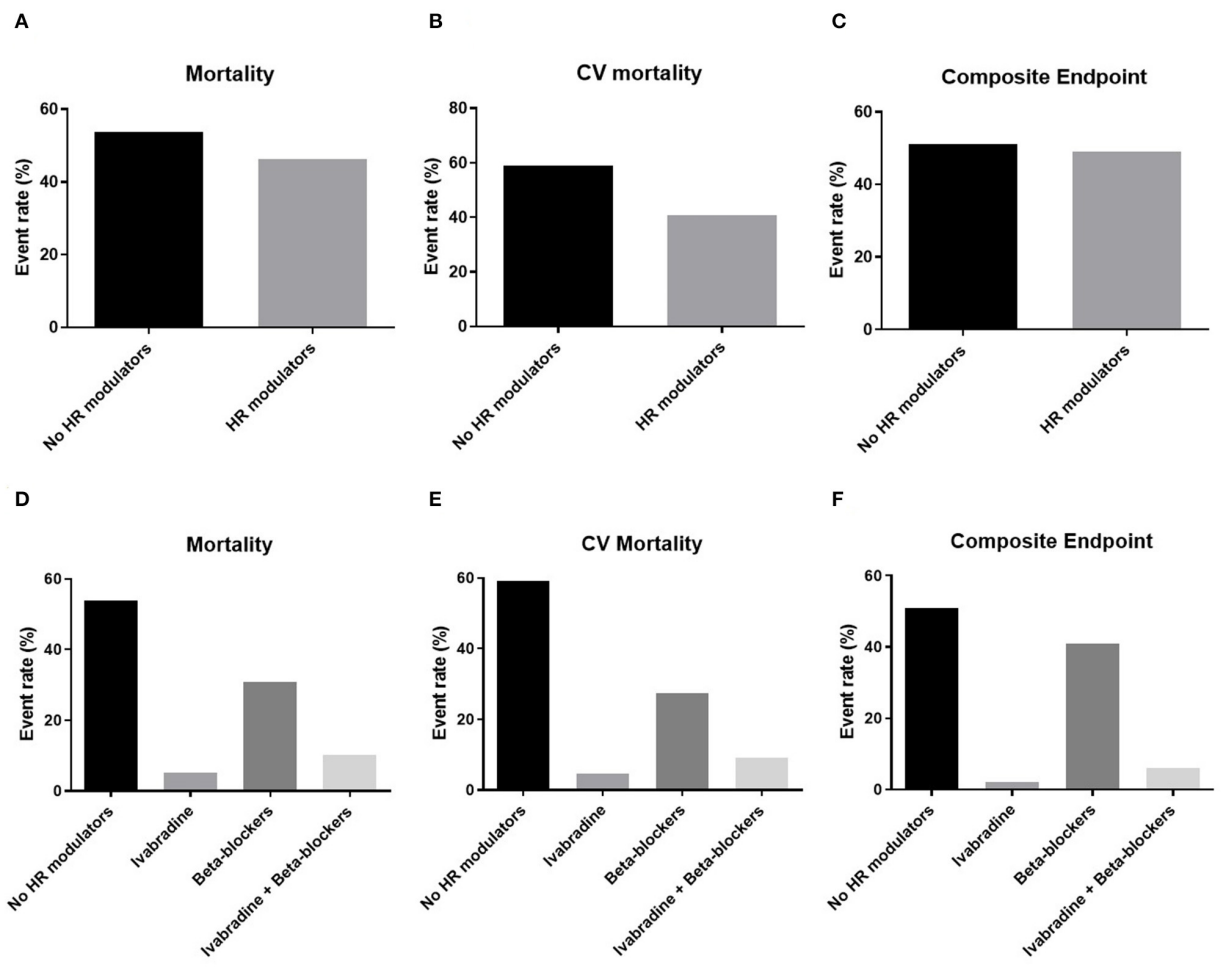
Event rates for mortality, cardiovascular (CV) mortality, and the composite endpoint of CV mortality or re-hospitalization for heart failure related to patients therapy. Top panels: the event rates for **(A)** all-cause mortality (Mortality), **(B)** cardiovascular mortality (CV mortality), and **(C)** the composite endpoint of CV mortality or re-hospitalization for HF (Composite endpoint) in the groups administered with and without heart rate (HR) modulators. Bottom panels: the event rates for **(D)** Mortality, **(E)** CV mortality, and **(F)** the Composite endpoint are displayed for patients administrated a therapy of either ivabradine or beta blockers only and a combined therapy of ivabradine and beta blockers.

Sixty-four patients (32%) were readmitted to the hospital, of which 27 (43%) for HF decompensation. Thirty-nine patients died (16%), of which 22 (56%) for cardiovascular causes. Overall, 49 patients met the conditions for the composite endpoint of cardiovascular death or hospitalization for HF (Figure 1). The all-cause mortality, the cardiovascular mortality and the composite endpoint event rates were higher in the population not taking HR modulators compared to the population taking at least one HR modulator (Figures 3A–C). The event rates of patients administered either beta blockers or ivabradine, or a combination of the two, are displayed in Figures 3D–F. Even if the sample sizes are small, we can note that the event rates for all-cause mortality, cardiovascular mortality, and for the combined endpoint of patients on ivabradine or on a combination of ivabradine and beta blockers were the lower compared to the other groups (Figures 3D–F).

### Predictors of the Cardiovascular Mortality, Composite Endpoint, and All-Cause Mortality by Multivariate Analysis

Heart rate at discharge ≥90 bpm was the strongest independent predictor of cardiovascular mortality (*OR* = 8.47; *p* = 0.016). The other parameter found to independently predict cardiovascular mortality was a history of revascularization performed during hospitalization (*OR* = 3.56; *p* = 0.021), while the presence of beta blockers represented again a protective factor (*OR* = 0.20; *p* = 0.004).

History of revascularization performed during the hospitalization (*OR* = 3.27; *p* = 0.007) and an LVEF > 50% (*OR* = 2.97; *p* = 0.035) were found to independently predict the composite endpoint. The presence of beta blockers at discharge, instead, was found to be protective against the composite endpoint (*OR* = 0.35; *p* = 0.013). We then performed a stratification of the population based on age (above vs. below 75 years), given the major prognostic role played by age in HF. In patients above 75 years old, we found a history of neoplasia (*OR* = 3.77; *p* = 0.019) and a history of revascularization during hospital stay (*OR* = 3.14; *p* = 0.018) to be independent predictors of the composite endpoint.

Regarding all-cause mortality, the presence of beta blockers at discharge was identified as a protective factor (*OR* = 0.35; *p* = 0.012). No other parameters were found to significantly predict all-cause mortality. Tables 4, 5 report the univariate and multivariate analysis for cardiovascular and all-cause mortality stratified by age.

**Table 4:** Uni- and multivariable analysis for the composite endpoint at 6 months.

**Composite endpoint**	**Univariable analysis**			**Multivariable analysis**		
**Risk factor**	**Coef**.	**OR (95% CI)**	***p*-Value**	**Coef**.	**OR (95% CI)**	***p*-Value**
Neoplasia	0.83	2.30 (0.94–5.63)	0.069	0.69	2.00 (0.76–5.32)	0.163
Anemia	0.68	1.98 (0.98–4.03)	0.058	0.81	2.26 (0.99–5.14)	0.053
Revascularization	0.80	2.23 (1.03–4.81)	0.041	1.18	3.27 (1.37–7.78)	0.007
LVEF>50%	0.79	2.21 (0.87–5.65)	0.097	1.08	2.97 (1.08–8.17)	0.035
Beta blockers at discharge	−1.02	0.36 (0.17–0.76)	0.007	−1.04	0.35 (0.15–0.80)	0.013

*The respective coefficient, 95% confidential interval, and p-value are displayed.*

* *p-value < 0.05.*.

**Table 5:** Composite endpoint stratified by age (above or below 75 yo).

**Composite endpoint (Age <75 years)**	**Univariable analysis**			**Multivariable analysis**		
**Risk factor**	**Coef**.	**OR (95% CI)**	***p*-values**	**Coef**.	**OR (95% CI)**	***p*-values**
**A**.						
Symptomatic PAOD	1.32	3.78 (0.78–18.34)	0.099	1.15	3.18 (0.63–16.05)	0.162
NT-proBNP at discharge ≥1,000	1.03	2.82 (0.83–9.54)	0.096	0.90	2.47 (0.71–8.60)	0.154
**B**.						
Neoplasia	1.18	3.29 (1.13–9.55)	0.029	1.32	3.77 (1.24–11.45)	0.019
ICD	1.38	4.00 (0.85–18.84)	0.08	1.51	4.56 (0.91–22.81)	0.065
Revascularization	0.91	2.49 (1.02–6.11)	0.046	1.14	3.14 (1.22–8.09)	0.018

*The respective coefficient, 95% confidential interval (CI), and p-value are displayed.*

* *p-value < 0.05*.

## Discussion

In this multi-center, prospective observational study, we assessed the impact of HR modulation in a selected cohort of acutely decompensated HF patients. In patients who received a combination of ivabradine and beta blockers, the event rates for the composite endpoint, all-cause mortality as well as cardiovascular mortality were lower compared to the population receiving no HR modulator therapy or taking one HR modulator only. Heart rate as a risk predictor has been mostly investigated in clinical trials assessing stable chronic HF patients (23, 24). Our study suggests the role of HR as an independent predictor of cardiovascular mortality in acutely decompensated HF patients. Nonetheless, an optimal HR at discharge is still a matter of discussion. Currently, the overall tendency in the clinical daily routine is to discharge patients as soon as they are clinically stable, independently from HR. In the SHIFT trial, HFrEF patients with HR > 70 bpm have been associated with a higher cumulative risk of cardiovascular death or hospitalizations for HF (composite endpoint) (24). Moreover, HFrEF patients discharged from the hospital with the highest HR (≥ 87 bpm) were found to be at a two-fold higher risk for the primary composite endpoint than patients presenting a lower HR (24). In our study, the threshold of HR > 90 bpm at discharge was shown to independently predict cardiovascular mortality in an equally distributed proportion of HFrEF and Heart Failure with preserved ejection fraction (HFpEF) patients. In the CHARM trial, an increase in HR during follow-up compared to the previous outpatient clinic visit was found to be a significant predictor of events (23).

Accordingly, in our study, we showed that a reduction of HR at follow-up through the administration of either beta blockers or a combination of beta blockers and ivabradine improves patients' survival. In previous studies, ivabradine has been shown to reduce the risk of re-hospitalizations due to worsening HF (25), reverse cardiac remodeling, and improvement of life quality in patients with HFrEF and in sinus rhythm (11, 26). Nonetheless, ivabradine use in the everyday clinical management of HR is still limited. This holds particularly true for acutely decompensated HF patients since only a few studies have investigated this topic (7). In a small cohort of patients admitted for acute decompensated HF and HR >70 bpm, ivabradine has been shown as a safe and effective tool not only to reduce HR but also to improve the clinical status and reduce NT-proBNP levels (27). In our study, we observed a reduction of HR in HF patients treated with either beta blockers or a combination of beta blockers and ivabradine during the course of hospitalization. Of note, after a follow-up period of 6 months, the HR was lower than admission in all treated groups, demonstrating a sustained effect of HR modulators. In the randomized trial by Hidalgo et al., the combined effects of ivabradine and beta blockers were compared with the use of beta blockers alone during acute hospital admission (28). Heart rate at 28 days and at 4 months after discharge were significantly lower in the combination group, which was also characterized by a significantly higher LVEF and lower brain natriuretic peptide levels. However, no differences in re-hospitalization and mortality at 4 months were found (28). In our study, instead, we showed that patients treated with a combination of beta blockers and ivabradine at 6 months after hospitalization had a lower global and cardiovascular mortality than the group treated with one or no HR modulators. Previous studies underlined the role of HR as a predictor of outcome and therapeutic target, particularly in patients in sinus rhythm (13, 29). However, the relationship appears weaker in patients with AFib especially in a chronic HF condition (30, 31). In our study, 42% of the total population was affected by AFib of which 56% were HFpEF patients. While in the HFrEF population diagnosed with AFib the recent CASTLE-AF study clearly showed the role of catheter ablation for the prevention of death and re-hospitalizations (32), the same is not evinced yet from the literature for the HFpEF population suffering from AFib (33). Atrial fibrillation is generally expected to be more prevalent in the older population (34, 35) and this is confirmed in our study. Nonetheless, we found AFib to be an independent predictor of mortality in the group of patients younger than 75 years (Table 5). This might be explained by a higher risk profile of these patients for their age and the higher prevalence of HFrEF. Interestingly, AFib patients presented a significantly higher HR at discharge; however, an increased risk of major cardiac events was observed for an HR > 90 bpm independently of patients' rhythm. Moreover, none of the patients with AFib was treated with ivabradine.

The parameter of revascularization, defined as any type of coronarography or stenting performed during the hospital stay, was found to independently predict cardiovascular mortality in the overall population. In patients >75 years old, revascularization was found to predict both all-cause mortality and cardiovascular mortality. This result is in line with the recent ISCHEMIA-HF trial (36), showing that among patients with stable ischemic heart disease, routinely performed invasive intervention failed to reduce major cardiac events compared with optimal medical therapy. No benefit was observed regarding invasive therapy for all-cause mortality or cardiovascular mortality. Routinely invasive therapy was even associated with harm at 6 months, and this is in accordance with our above-mentioned results.

The data presented in the current work point toward the need to carefully evaluate an early in-hospital HR reduction in acutely decompensated HF patients, and we believe that this aspect is worth being investigated in larger studies in the future.

## Limitations

Our study has several limitations. From a relatively high number (37) of patients, we either did not receive information for building our database, or this was lost at follow-up. For this reason, we excluded them from the follow-up analysis, potentially underestimating the overall mortality rate. Moreover, a relatively low number of patients were administered with ivabradine alone, thus hampering a more accurate analysis of the effects of ivabradine on the HR. However, this was expected because the use of ivabradine for this selected cohort of patients is not common. Furthermore, the type of beta blockers as well as the average dose of beta blockers and ivabradine has not been collected consistently in the dataset and was therefore not included in the manuscript. The population examined was enrolled in both cardiology and internal medicine departments, holding a certain heterogeneity in patients handling. However, this heterogeneity does not represent the scope of the paper, and the authors of this study reserve the right to investigate it in a further study. Since the study was started in 2017, patients did not receive an ARNI treatment. In the current dataset, only a few patients were found to be on digitalis at admission (7%); however, even if no effect was observed in our risk prediction model, the data should be interpreted with caution since digitalis is known to reduce survival in HF patients (37). Eventually, residual confounding variables cannot entirely be excluded. Of note, we did not investigate the adherence of patients to HR modulatory medications.

## Conclusions

In conclusion, higher HR at discharge is associated with cardiovascular mortality at 6 months but not with the composite endpoint of cardiovascular death or re-hospitalization for HF. Therapy with a combination of heart-rate modulators, such as ivabradine and beta blockers, is associated with lower event rates in our cohort of patients.

## Data Availability Statement

The raw data supporting the conclusions of this article will be made available by the authors, without undue reservation.

## Ethics Statement

The study was conducted according to the directions of the Declaration of Helsinki (1964) with the approval of the local Ethical Committees of Ospedale Sant'Anna di Como and acquiring patients' informed consent. The patients/participants provided their written informed consent to participate in this study.

## Author Contributions

CC, AF, GDT, CDC, DN, and MG were involved in study design, planning, and patients recruitment and follow-up. AA, DA, GC, and KR processed the experimental data, performed the analysis, drafted the manuscript, and designed the figures. AA and CC aided in interpreting the results and worked on the manuscript. All authors discussed the results and commented on the manuscript.

## Conflict of Interest

AF is a shareholder of the Company BOCAhealthcare GmbH. BP reports having received consultancy and lecture honoraria from Bayer, Daiichi Sankyo, MSD, Novartis, Sanofi-Aventis, Stealth Peptides, and Vifor Pharma; and editor honoraria from the Journal of the American College of Cardiology. BP and SK received funding from the DZHK (German Centre for Cardiovascular Research) and by the BMBF (German Ministry of Education and Research). SK received an unrestricted research grant from Philips Healthcare and received lecture honoraria from Medis, NL. The remaining authors declare that the research was conducted in the absence of any commercial or financial relationships that could be construed as a potential conflict of interest.

## Publisher's Note

All claims expressed in this article are solely those of the authors and do not necessarily represent those of their affiliated organizations, or those of the publisher, the editors and the reviewers. Any product that may be evaluated in this article, or claim that may be made by its manufacturer, is not guaranteed or endorsed by the publisher.
